# Irrigation Ponds as Sources of Antimicrobial-Resistant Bacteria in Agricultural Areas with Intensive Use of Poultry Litter

**DOI:** 10.3390/antibiotics11111650

**Published:** 2022-11-18

**Authors:** Eliene S. Lopes, Cláudio E. T. Parente, Renata C. Picão, Lucy Seldin

**Affiliations:** 1 Laboratório de Genética Microbiana, Instituto de Microbiologia Paulo de Góes, Universidade Federal do Rio de Janeiro (UFRJ), Rio de Janeiro 21941-902, Brazil; 2 Laboratório de Radioisótopos Eduardo Penna Franca, Instituto de Biofísica Carlos Chagas Filho, Universidade Federal do Rio de Janeiro (UFRJ), Rio de Janeiro 21941-902, Brazil; 3 Laboratório de Investigação em Microbiologia Médica, Instituto de Microbiologia Paulo de Góes, Universidade Federal do Rio de Janeiro (UFRJ), Rio de Janeiro 21941-902, Brazil

**Keywords:** poultry litter, antimicrobial resistance genes, bacterial community, irrigation ponds, fluoroquinolones, β-lactams, sulfonamides

## Abstract

Poultry litter is widely used worldwide as an organic fertilizer in agriculture. However, poultry litter may contain high concentrations of antibiotics and/or antimicrobial-resistant bacteria (ARB), which can be mobilized through soil erosion to water bodies, contributing to the spread of antimicrobial resistance genes (ARGs) in the environment. To better comprehend this kind of mobilization, the bacterial communities of four ponds used for irrigation in agricultural and poultry production areas were determined in two periods of the year: at the beginning (low volume of rainfall) and at the end of the rainy season (high volume of rainfall). 16S rRNA gene sequencing revealed not only significantly different bacterial community structures and compositions among the four ponds but also between the samplings. When the DNA obtained from the water samples was PCR amplified using primers for ARGs, those encoding integrases (*intI1*) and resistance to sulfonamides (*sul1* and *sul2*) and β-lactams (*bla*_GES_, *bla*_TEM_ and *bla*_SHV_) were detected in three ponds. Moreover, bacterial strains were isolated from CHROMagar plates supplemented with sulfamethoxazole, ceftriaxone or ciprofloxacin and identified as belonging to clinically important Enterobacteriaceae. The results presented here indicate a potential risk of spreading ARB through water resources in agricultural areas with extensive fertilization with poultry litter.

## 1. Introduction

Antimicrobial resistance (AMR) is a global public health problem that generates social and economic impacts [1,2]. Although AMR is a natural evolutionary phenomenon, its main driver is the widespread use of antimicrobials in human and veterinary medicine [3].

In animal husbandry, it is estimated that the use of antimicrobials will increase by 70% between 2010 and 2030 with the intensification of production to meet the growing demand for cheap animal protein [4,5,6]. The poultry industry draws attention to the wide use of antimicrobials, such as β-lactams, sulfonamides, and fluoroquinolones, which are widely used due to their broad spectrum against Gram-positive and Gram-negative pathogens [7,8,9]. The use of antimicrobial agents as growth promoters is controlled in animal husbandry in Brazil. However, many molecules may still be used for therapeutic, prophylactic and metaphylactic purposes [4,10]. This constant exposure to antimicrobials, especially in subtherapeutic doses, may result in selective pressure on the poultry microbiota. Therefore, increased expression and transfer of antimicrobial resistance genes (ARGs) from one organism to another, triggering a reduction in the sensitivity of microorganisms to these drugs, can be observed [11].

Additionally, most antimicrobials are not completely metabolized by the animal organism, and bioactive compounds are excreted without changes. These bioactive compounds are able to reach the environment mainly through the application of poultry litter in agricultural soils [12,13]. Poultry litter, mainly composed of chicken manure, spilled feed, feathers, and bedding materials, is the main waste of poultry farming, and millions of tons are generated annually worldwide, providing macronutrients and improving the physical, chemical and biological characteristics of soils [14]. However, considering the risks to public health, it is necessary to comprehensively verify the environmental fate of contaminants due to poultry litter fertilization in agricultural environments [15].

Previous studies have reported that antimicrobials can accumulate in soils and reach surface and groundwater through erosive processes, such as leaching and surface runoff, increasing selective pressure in these environments [12,16,17,18,19]. Contaminated water can be an important route of transmission of pathogens through animal watering, human supply, and irrigation of agricultural crops [20,21].

In this context, to better understand the influence of the use of poultry litter on the spread of antimicrobial-resistant bacteria (ARB) and antimicrobial resistance genes (ARGs) and to predict possible risks to human health, the bacterial communities found in the water of four irrigation ponds surrounded by agricultural areas with intensive use of poultry litter were characterized. Moreover, the presence of clinically important ARB and the occurrence of β-lactam, sulfonamide and fluoroquinolone resistance genes were also investigated. As the study area is situated in a highly weathered tropical region (average annual rainfall greater than 2500 mm [22,23], and considering that the wet season is the period of the greatest mobilization (soil–water) of contaminants (through soil leaching and surface water runoff), two periods of the year—high and low rainfall (end and beginning of rainy season, respectively)—were evaluated to understand seasonal influences on the evaluated bacterial communities.

## 2. Materials and Methods

### 2.1. Study Area and Sampling Design

The study area comprises agricultural areas in the municipality of São José do Vale do Rio Preto (SJVRP; 220 km^2^ and 615 m.a.s.l. mean elevation), located in the upland region of Rio de Janeiro state, in southeastern Brazil (Figure S1, [24,25]). The climate of the region is classified as Altitude Tropical, with dry winters and humid summers [22,23].

The municipality stands out as the largest poultry producer in Rio de Janeiro state (RJ), housing approximately one hundred poultry farms surrounded by agricultural areas that supply fresh products to the RJ metropolitan region [24]. In SJVRP, agricultural soils are periodically fertilized with poultry litter contaminated with veterinary antimicrobials [24,25] and irrigated with water from surrounding ponds. The relief features with slopes and agricultural valleys can favor erosive processes, such as leaching and surface runoff along drainage basins, reaching irrigation ponds. The wet season is considered the critical period for the mobilization (soil–water) of contaminants. Therefore, two samplings (denoted S1 and S2) were carried out in the wet season to assess periods with high (S1; on 14 March 2019) and low monthly rainfall (S2; on 25 November 2019). The amount of rain was significantly different between samplings: S1 (187 mm) and S2 (107 mm) [26].

Water samples were collected in four artificial ponds used for crop irrigation: LA (22°11′35.4″ S, 42°55′11.0″ W) with 245 m^2^; LB (22°11′31.8″ S, 42°55′06.3″ W) with 147 m^2^; LC (22°06′52.7″ S, 42°57′04.2″ W) with 665 m^2^ and LD (22°08′57.4″ S, 42°52′54.4″ W) with 294 m^2^ (Figure S2). The main crops in the surroundings were chayote, tomato, zucchini, bell pepper, among others.

The water samples (1 L) were collected (up to 30 cm deep) in triplicate with distances ranging from 1 to 10 m between each replicate in sterile glass bottles. They were identified by capital letters followed by the number of replicates: LA (LA1, LA2 and LA3), LB (LB1, LB2 and LB3), LC (LC1, LC2 and LC3) and LD (LD1, LD2 and LD3).

The physicochemical parameters of the pond waters—pH, salinity, and temperature—were measured using a portable meter (Mettler Toledo, Inc., Schwerzenbach, Switzerland). These data are shown in Table S1.

### 2.2. DNA Extraction from Water Samples

For the extraction of total DNA, 250 mL of each water sample was filtered through Millipore filters with 0.45 µm pore size membranes using a Kitassato connected to a vacuum pump. DNA extraction from water samples was performed from the membranes using the FastDNA Spin kit (MP Biomedicals, Santa Ana, CA, USA) according to the protocol provided by the manufacturer. After extraction, the concentration of the obtained DNA samples was evaluated using a Qubit 4 fluorometer (Thermo Fisher Scientific, Waltham, MA, USA).

### 2.3. Detection of β-Lactam, Sulfonamide and Fluoroquinolone Resistance Genes

Simplex and multiplex PCRs were performed using the DNA extracted from the four ponds to detect genes encoding class 1 and 2 integrases (*intI1* and *intI2*) and genes encoding resistance to β-lactams (*bla*_CTX-M_, *bla*_TEM_, *bla*_SHV_, *bla*_GES_, *bla*_FOX-like_, *bla*_MOX-like_, *bla*_CIT-like_, *bla*_EBC-like_, *bla*_DHA-like_, *bla*_ACC-like_, *bla*_MIR_ and *bla*_ACT_), sulfonamides (*sul1* and *sul2*) and fluoroquinolones (*qnrA*, *qnrS*, *qnrC*, *qnrD*, *qnrB*, *qnrVC* and *qepA*). The primer sequences used and the amplification conditions are presented in Table S2 [27,28,29,30,31,32,33,34]. The PCR products were visualized after electrophoresis in a 1.4% agarose gel in 1X TBE buffer [35]. The presence or absence of common bands among the different ponds was compared and presented in a Venn diagram.

### 2.4. Amplicon Sequencing of 16S rRNA Genes from Water Samples

Total DNA extracted (approximately 10–20 ng/μL) from the 24 samples (four ponds in triplicate and two samplings) was sequenced using the MiSeq system (Illumina, USA) paired-end by Novogene Corporation (Beijing, China). The primers used for PCR amplification of the V4 region of the *rrs* gene (encoding 16S rRNA) were 515F (GTGCCAGCMGCCGCGGTAA) and 806R (GGACTACHVGGGTWTCTAAT) [36,37], which generated fragments of approximately 250 bp.

### 2.5. Bioinformatics Analysis

The sequences obtained from sequencing were analyzed using Mothur v.1.44.3 software [38]. The forward and reverse sequences were paired in contigs, and sequences with sizes incompatible with these fragments, ambiguities and homopolymers (>8) were removed from the analysis, while similar sequences were grouped to eliminate redundancies. Virtual PCR was performed using primers 515F and 806R [36,37] to align the consensus sequences based on the Silva v138 database [39]. Sequences with insufficient alignments and columns without nucleotides were removed. A precluster was performed for error correction in rare sequences. The sequences were classified using the Ribosomal Database Project (RDP) [40] to remove contaminants such as mitochondrial DNA, chloroplasts, Archaea, Eukarya, chimeric sequences and nontaxon organisms. The Bayesian method was used for the classification of sequences based on the RDP dataset [40]. Using a cutoff of 97% similarity, the sequences were grouped into operational taxonomic units (OTUs), and singletons were removed. Finally, the data related to *α*-diversity and β-diversity indexes, rarefaction curves, and taxonomic relative abundance were used in further statistical analyses.

Raw sequence data were deposited in the NCBI Sequence Read Archive (SRA) and are available under Bioproject accession number (PRJNA895046).

### 2.6. Statistical Analyses

The statistical analyses of sequencing data were performed in Past v4.03 software [41] based on two independent variables: the sampling period (March and November 2019, corresponding to the first (S1)—high rainfalls—and second (S2)—low rainfalls—samplings, respectively) and the area (the four studied ponds). The richness and diversity indexes, as well as the relative abundance of the taxa (phylum, class and genus), were checked for distribution normality by the Shapiro–Wilk test or homoscedasticity among samples by the Levene test. The resulting data were submitted to normalization by Box Cox [42] when necessary. Student’s *t* test was applied to parametric data, while the Mann–Whitney test was applied to nonparametric data to compare the bacterial communities between the two samplings from each pond. Parametric data were submitted to one-way analysis of variance (ANOVA), and the nonparametric data were submitted to the Kruskal–Wallis test to compare the bacterial communities of the four ponds at each sampling. In addition, richness and diversity indexes were also submitted to ANOVA.

Finally, nonmetric multidimensional scaling (nMDS) was performed with the Bray–Curtis dissimilarity index using the OTU distribution matrix followed by two-way PERMANOVA to test the distribution of OTUs considering the sampling periods and the ponds studied. The physical–chemical variables (pH, salinity and temperature) were integrated into nMDS as metadata.

### 2.7. Isolation of ARB from Water Samples

Bacterial strains resistant to different antimicrobials were isolated from water samples in the second sampling campaign (S2, low rainfall sampling). The triplicates were mixed (750 mL of water from each replicate), and 1000 mL of water from each pond was filtered through 0.45 μm pore size Millipore^®^ (Barueri, SP, Brazil) membranes using a Kitassato connected to a vacuum pump. The four membranes were washed individually in 20 mL of saline (NaCl 0.85%).

Aliquots of 100 µL of each sample and their respective dilutions (10^−1^ and 10^−2^) were plated in Petri dishes containing CHROMagar Orientation culture medium (BD Diagnostics) supplemented with 50 μg/mL ciprofloxacin (Sigma^®^, Saint Louis, MI, USA), 60 μg/mL sulfamethoxazole (Sigma^®^, Buchs, Switzerland) or 8 μg/mL ceftriaxone (Sigma^®^ Saint Louis, MI, USA) [43,44]. The plates were incubated at 37 °C for 24 h. After determination of colony forming units per milliliter (CFU/mL), three colonies of each morphology were reinoculated on CHROMagar and further identified at the genus level by MALDI-TOF (Bruker Daltonics Bremen, Germany). All isolated strains were maintained at −80 °C in tryptic soy broth medium (TSB) supplemented with the antibiotic used for their isolation, and 20% glycerol.

## 3. Results

### 3.1. PCR Amplification of Antimicrobial Resistance Genes in Water Samples

**Figure 1:**
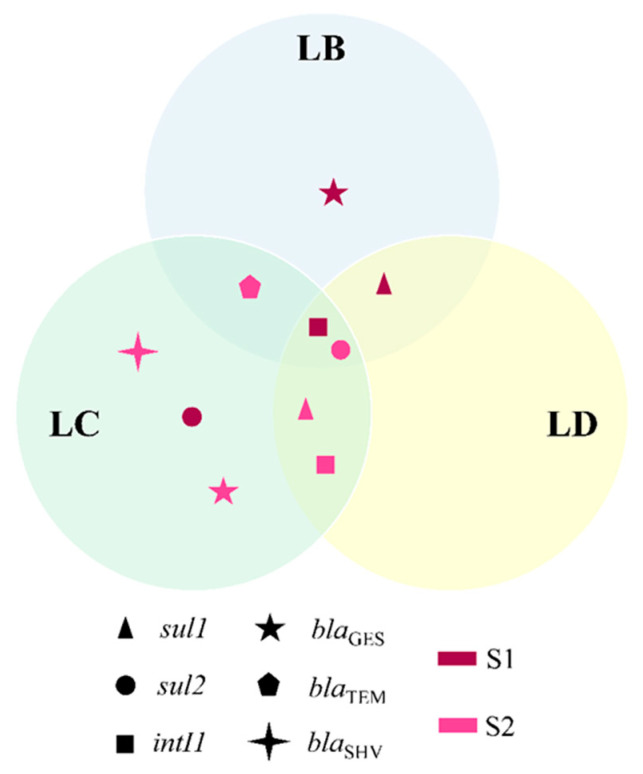
Presence of resistance genes in the studied ponds. Resistance genes are represented by geometric figures—dark pink in S1 and light pink in S2—in a Venn diagram. S1 and S2 correspond to the sampling period: high (S1; on 14 March 2019) and low monthly rainfall (S2; on 25 November 2019).

After DNA extraction from the water samples of the four ponds studied here in two periods of the year corresponding to high (S1) and low (S2) rainfall samplings, β-lactams resistance genes were detected in two ponds (LB and LC). The genes *bla*_GES_ and *bla*_TEM_ were detected in LB in S1 and S2, respectively, while both genes and the *bla*_SHV_ gene were also observed in LC, but only in S2 (Figure 1 and Figure S3A–C).

Sulfonamide resistance genes were detected in three ponds (LB, LC and LD). Bands corresponding to *sul1* were observed in LB and LD (S1) and in LC and LD (S2), while bands corresponding to *sul2* were observed in LC (S1) and in LB, LC and LD (S2) (Figure 1 and Figure S4A,B).

Genes encoding integrases were also detected in three of the four ponds (LB, LC and LD). Bands corresponding to the *intI1* gene were observed in the LB, LC and LD (S1) and in the LC and LD (S2) (Figure 1 and Figure S5A).

Only in one pond (LA) was the presence of the abovementioned genes not observed. Furthermore, amplification of genes encoding resistance to fluoroquinolones (*qnrA*, *qnrS*, *qnrC*, *qnrD*, *qnrB*, *qnrVC* and *qepA*) was not detected in any of the studied ponds.

### 3.2. Structure and Composition of the Bacterial Communities of Water Samples

After sequencing the V4 region of the gene encoding 16S rRNA of the 24 DNA samples obtained from the four ponds, the sequences were analyzed and normalized to 47,990 sequences per sample. The number of sequences represented a sufficient coverage of the OTUs present in the bacterial communities, which can be observed by the tendency of the rarefaction curves to reach a plateau (Figure S6).

**Figure 2:**
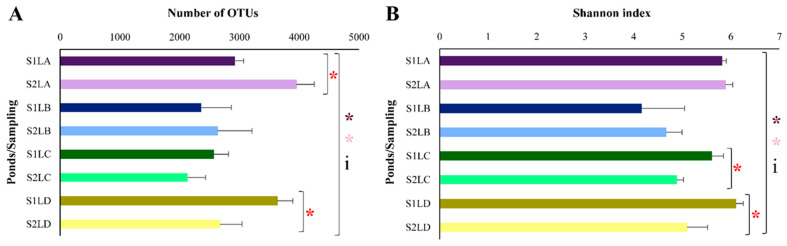
Alpha diversity evaluated by massive sequencing of the gene encoding 16S rRNA in water samples collected in LA (purple), LB (blue), LC (green) and LD (yellow) in S1 (high rainfall period—dark tones) and in S2 (low rainfall period—light tones). The bars represent the standard deviation. Species richness and alpha diversity showed significant differences between the ponds in the two samplings (parametric data submitted to a one-way ANOVA) represented by asterisks (dark pink for S1 and light pink for S2). A red asterisk represents the significant difference among the ponds at each sampling (parametric data submitted to the t test). The data were also submitted to a two-way ANOVA, and the interaction between the factors is represented by the letter i. (**A**) Richness evaluated by the number of OTUs; (**B**) Shannon diversity index.

A significant difference was observed for richness (number of OTUs) between S1 and S2 for ponds LA (*p* = 0.0058288) and LD (*p* = 0.021009) (Figure 2A). Statistical differences were also observed in either S1 (*p* = 0.00584) or S2 (*p* = 0.003146) among the four ponds (Figure 2A). Considering the Shannon diversity index (Figure 2B), significant differences between S1 and S2 for LC (*p* = 0.011584) and for LD (*p* = 0.018024) were observed among the four ponds in S1 (*p* = 0.004127) and S2 (*p* = 0.004021). Moreover, an interaction between sampling periods (S1 and S2) and ponds (four water samples), related to both richness (*p* = 0.001341) (Figure 2A) and diversity (*p* = 0.01374) (Figure 2B), was observed using a two-way ANOVA.

**Figure 3:**
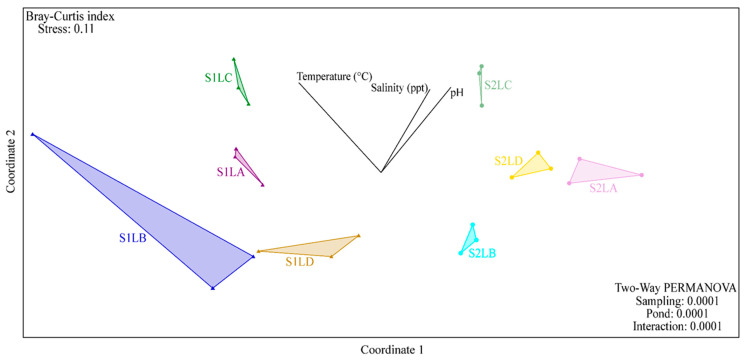
Nonmetric multidimensional scaling (nMDS) with the Bray–Curtis dissimilarity index. Representation of LA (purple), LB (blue), LC (green) and LD (yellow). The replicates are represented by triangles in dark tones (S1) and circles in light tones (S2). The physical–chemical data evaluated from the water samples from the ponds were integrated as metadata.

Beta diversity was evaluated by nonmetric multidimensional scaling (nMDS) of OTUs based on the Bray–Curtis dissimilarity index (Figure 3). From a two-way PERMANOVA, it was observed that the structure of the bacterial communities of each pond was different between samplings (S1 and S2) (*p* = 0.0001). Additionally, a clear dispersion among the four ponds (*p* = 0.0001) was observed (Figure 3). With the integration of the physicochemical data to the NMDs, the temperature positively influenced the first sampling (S1) of LC, while pH and salinity positively influenced the second sampling (S2) of this pond (Figure 3).

**Figure 4:**
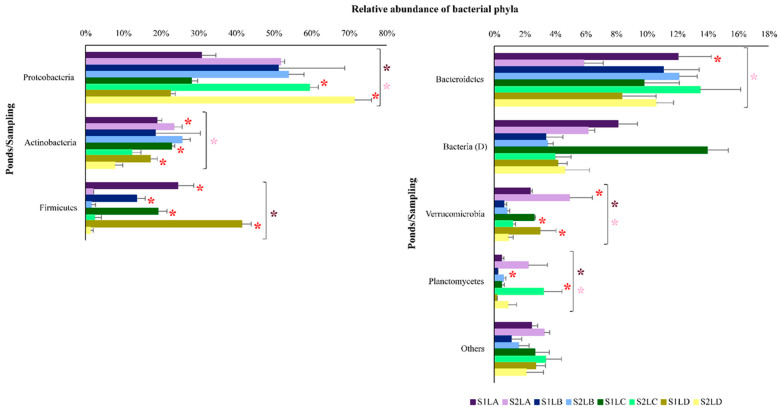
Relative abundance of bacterial phyla in water samples collected in LA (purple), LB (blue), LC (green) and LD (yellow) in S1 (high rainfall period—dark tones) and in S2 (low rainfall period—light tones). The bars represent the standard deviation. Asterisks (dark pink for S1 and light pink for S2) represent the significant differences among the ponds in the two samplings (parametric data submitted to a one-way ANOVA and nonparametric data submitted to Kruskal–Wallis). A red asterisk corresponds to the significant difference between the samples of each pond (parametric data submitted to the t test and nonparametric data submitted to Mann–Whitney). (D) next to the bacterial taxon means that the classification was at the domain level, and no statistical analysis was applied. The data represented by “others” were also not statistically analyzed because they are formed by more than one taxon.

The relative abundance of bacterial taxa in the four water samples was determined in S1 and S2. All phyla with at least 1% of the total relative abundance are shown in Figure 4. A significant difference was observed among the ponds in the relative abundance of Proteobacteria, Verrucomicrobia and Planctomycetes in S1 and S2. For Firmicutes, a significant difference among the ponds was observed only in S1, while for Actinobacteria and Bacteroidetes, a significant difference was observed only in S2. In addition, the following can be observed in Figure 4: (i) a significant difference in the relative abundance of Proteobacteria between S1 and S2 in LC and LD; (ii) a significant difference in the abundances of Actinobacteria and Verrucomicrobia between S1 and S2 in all water samples except in LB; (iii) the predominance of Actinobacteria and Verrucomicrobia in LC and LD (in S1) and in LA (in S2); and (iv) the predominance of Firmicutes in the four ponds in S1 and Bacteroidetes in LA (S1).

At the class level, the relative abundance of the taxa also varied among the four ponds and between the two samplings (Figure S7). Significant differences were observed in S1 and S2 in the relative abundance of classes Betaproteobacteria, Alphaproteobacteria, Clostridia, Sphingobacteria, Gammaproteobacteria, Cytophaga, Deltaproteobacteria and Planctomycetia. The predominance of Actinobacteria and Bacilli varied among the ponds in S2 and S1, respectively. In addition, between the two samplings of the four ponds, Clostridia was the only class that predominated in S1. Ten classes showing relative abundances of at least 1% are represented in Figure S7.

More than 40% of the sequences were taxonomically classified at the genus level. A significant difference was observed among the four ponds in the relative abundance of the genera *Clostridium*, *Sediminibacterium*, *Pseudarcicella* and *Polynucleobacter* in both samplings. *Clostridium* was predominant in the four ponds in S1. The genera *Paenibacillus* and *Methylocystis* showed significant differences only in S1. The genus *Paenibacillus* was more prevalent in LC and LD (in S1). In S2, *Rhodobacter* was more abundant in LC, and the relative abundance of *Acidovorax*, *Novosphingobium* and *Rhodobacter* varied in the four ponds (Figure S8).

### 3.3. Strains of ARB Identified by MALDI-TOF

A total of 135 bacterial strains isolated from CHROMagar plates supplemented with the different antimicrobials were submitted to MALDI-TOF identification. From this total, 102 strains showed score values between 1700 and 2448.

No growth was observed from LC in cultures supplemented with ciprofloxacin. The highest number of colonies was observed in LA (8 × 10^2^ CFU/mL). Three colonies of each morphology observed in the different plates (from LA, LB and LD) were reinoculated in ciprofloxacin-supplemented plates, but only three of these strains (isolated from LD) could be identified as belonging to the genus *Escherichia*, with score values between 1961 and 2238 (Table S3).

Fifty-four bacterial strains recovered from cultures supplemented with ceftriaxone were identified with score values between 1700 and 2402: 10 strains of LA; 11 strains of LB; 23 strains of LC and 10 strains of LD. Strains belonging to the genus *Chryseobacterium* were identified from samples from all four ponds. On the other hand, strains belonging to the genus *Elizabethkingia* were observed only in LB, *Acinetobacter* sp. and *Stenotrophomonas* sp. only in LC, while *Enterobacter* sp. and *Proteus* sp. only in LD (Table S4).

Forty-five strains recovered from sulfamethoxazole-supplemented cultures were identified with score values between 1700 and 2438: 14 of LA; 8 of LB; 14 of LC and 9 of LD. Strains belonging to the genera *Escherichia* and *Klebsiella* were isolated from all ponds, while strains of *Aeromonas* and *Cronobacter* were identified only in LA, and those belonging to the genus *Serratia* were identified only in LB. Strains belonging to the genera *Enterobacter* and *Proteus* were found in LA, LC and LD (Table S5).

### 3.4. Comparison of Bacterial Community Composition with the Prevalence of ARB

By sequencing the gene encoding 16S rRNA, different OTUs could be related to genera attributed to bacterial strains resistant to the different antimicrobials tested and identified by MALDI-TOF (Figure S9).

In general, isolated strains identified as belonging to the genera *Pseudomonas*, *Bacillus*, *Acinetobacter*, *Stenotrophomonas*, *Chryseobacterium*, *Aeromonas*, *Pantoea*, *Escherichia* and *Citrobacter* were also found in the taxonomic classification of the different OTUs in all ponds. Bacterial strains from other genera belonging to the Enterobacteriaceae family were also identified, such as *Cronobacter*, *Enterobacter*, *Proteus* and *Klebsiella*.

A significant difference was observed among the ponds (one-way ANOVA/Kruskal–Wallis) in the relative abundance of OTUs associated with the genera *Pseudomonas* and *Bacillus* in S1 and with *Aeromonas* and Enterobacteriaceae in S2. Statistical differences were also observed between S1 and S2 in the relative abundance of *Bacillus* and *Pantoea* in LC and of Enterobacteriaceae and *Bacillus* in LD (Student’s *t* test/Mann–Whitney).

### 3.5. ARGs in Isolated Strains

Forty-four isolated strains were PCR amplified for the presence of ARGs using specific primers. Table 1 shows all strains where a PCR product was detected. Among the strains grown on ceftriaxone plates, *Escherichia* strains (isolated from LA) were positive for the *bla*_TEM_ gene. Strains of *Klebsiella* (isolated from LA) were positive for *bla*_TEM_ and *bla*_SHV_ (Table 1, Figure S3B). Among the strains previously isolated on sulfamethoxazole-containing plates, the *sul1* gene was detected in an *Enterobacter* strain (isolated from LA) (Table 1, Figure S4A) and the *sul2* gene was detected in strains belonging to the genera *Escherichia*, *Proteus* and *Aeromonas* (isolated from LA and LD) (Table 1, Figure S4B). The *intI1* gene was observed in *Escherichia* and *Enterobacter* strains (isolated from LA and LD) (Table 1, Figure S5), and the *intI2* gene was observed in *Enterobacter* and *Pantoea* strains (isolated from LA and LB) (Table 1, Figure S3).

## 4. Discussion

It is well known that surface waters act as a sink for pollutants from terrestrial environments. Therefore, there is a great concern regarding the fate of antimicrobials and the role of bacterial communities in the dissemination of antimicrobial resistance genes in water sources. Several studies have demonstrated the presence of antibiotics, families of ARGs and ARB in aquatic ecosystems, including wastewaters, sea water, surface water (rivers, ponds, and lakes), recreational or drinking water [45,46,47,48,49,50,51]. This confirms the widespread dissemination of resistance in aquatic systems and the importance of the problem worldwide [49]. However, studies with this approach are still incipient not only in Brazil but also in other countries, especially when surface water in agricultural producing areas with the use of poultry litter is considered [52,53].

In this study, β-lactams resistance genes (*bla*_GES_, *bla*_TEM_ and *bla*_SHV_) were detected in two ponds (LB and LC). Furlan and Stehling (2018) [54] also detected the *bla*_SHV_ gene in water, feces, and soil from a pig farm in Brazil. However, the *bla*_GES_ gene could not be detected in the water samples analyzed. Indeed, GES β-lactamases (encoded by *bla*_GES_) are less frequently found than other ESBLs (Extended Spectrum β-lactamases) and carbapenemases, except for bacterial strains isolated from clinical settings [54,55]. Therefore, the presence of the *bla*_GES_ gene in the ponds studied here corroborates the data obtained by Jurelevicius et al. (2021) [51], where this gene and the *bla*_TEM_ gene were observed in marine waters.

Sulfonamide resistance genes and genes encoding integrases were simultaneously detected in the three ponds. The detection of *sul1* and *intI1* genes in the same sample is not a surprise, as the *sul1* gene is often found in the conserved regions of class 1 integrons. Therefore, in addition to the potential risk of accumulation of *sul1* genes, this result may indicate the risk of dissemination of the *sul* genes by the frequent association of integrons with transposons and plasmids [56,57]. Lai et al. (2021) [50] also observed similar results in surface water sources in Sweden and highlighted the importance of the *intI1* and *sul1* genes as genetic markers of anthropogenic pollution. The *sul1* and *sul2* genes were also the most prevalent genes detected by PCR in river water samples from Germany and Australia, showing the environmental spread of these genes worldwide [45].

Although fluoroquinolone resistance genes were not detected on the water surface of the ponds in this study, we cannot exclude the possibility of their presence in a small amount, below the detection limit of the PCR technique. The occurrence of the *qnrS* gene and high concentrations of ciprofloxacin and enrofloxacin (fluoroquinolones) have already been reported in agricultural soils from the same region [24]. Moreover, fluoroquinolones have a low leaching potential and are usually detected in water at low concentrations, as they are strongly adsorbed to soils and sediments [58,59]. In contrast, sulfonamides have a high leaching potential and have been detected in high concentrations on water surfaces [60,61]. Vollú et al. (2018) [14] did not detect the *qnrA*, *qnrB* and *qnrS* genes in poultry litter samples in the presence of enrofloxacin and ciprofloxacin (fluoroquinolones) residues. The authors suggested that either these genes were not present or were present in low copy numbers, below the detection limit of the PCR technique.

The differences observed in the structure and composition of the bacterial communities in the four ponds may be related to soil fertilization and the amount of precipitation (which was significantly different between samplings), in addition to the physicochemical characteristics of the water samples. The phyla Firmicutes, Clostridia (class) and *Clostridium* (genus) were significantly more abundant in S1. Likewise, Clostridia has been described as an abundant class in the microbiota of poultries that are treated with penicillins [62,63]. Considering that S1 occurred in the wet season in a period of high rainfall volume, it is possible that bacteria of these taxa were mobilized from the soil fertilized with the poultry litter to the ponds by the surface runoff of water and leaching. Brooks et al. (2009) [62] reported that the species *Clostridium perfringens* can be an indicator of fecal contamination and of mobilization of poultry litter by surface runoff.

When ARB were isolated from the different ponds and further characterized, strains identified as enterobacteria (*Escherichia* sp., *Enterobacter* sp., *Aeromonas* sp., *Proteus* sp. and *Pantoea* sp.) were isolated from all sulfamethoxazole-containing plates. One of the most common mechanisms of sulfonamide resistance in enterobacteria is the acquisition of *sul* genes, which encode resistant variants of the dihydropteroate synthase (DHPS) enzyme. Usually, *sul1* and *sul2* genes are found among sulfonamide-resistant Enterobacteriaceae with the same frequency [64,65]. Considering the results obtained here using primers for *sul1*, *sul2*, *intI1* and *intI2* genes, there is a potential risk of dissemination of sulfonamide resistance among clinically important bacteria in the studied ponds.

In the same way, as genes encoding ESBLs were detected in water samples (*bla*_GES_, *bla*_SHV_ and *bla*_TEM_) and in *Klebsiella* and *Escherichia* strains, there is a potential risk of dissemination of β-lactam resistance in these environments, especially considering that the genes *bla*_SHV_ and *bla*_TEM_ are plasmid-mediated and often reported in these genera. These genes encode narrow-spectrum β-lactamases or ESBLs that hydrolyze broad spectrum cephalosporins and monobactams [66]. The production of these enzymes by pathogenic strains of *E. coli* and *K. pneumoniae* has been reported by researchers worldwide, and their prevalence has been increasing from 6% to 88% in healthcare settings [66,67,68]. The presence of these ARGs in surface water sources indicates the dissemination of AMR in the environment. Elshafiee et al. (2022) [69] also detected the presence of *bla*_SHV_ and *bla*_TEM_ genes in *K. pneumoniae* isolates in irrigation waters from fresh produce farms in Egypt. Likewise, Amato et al. (2021) [49] also detected a high prevalence of *bla*_TEM_ in *E. coli* strains isolated from irrigation water surface sources in Spain. These studies indicate the risk of transmission of resistant β-lactams for farmers and through the consumption of commercialized products. Bacteria of the Enterobacteriaceae family are often associated with intra-abdominal infections and can be transmitted by contaminated food and water, such as pathogenic strains of *E. coli* and *K. pneumoniae* that can cause gastrointestinal and urinary tract infections (UTIs) [69,70].

Finally, some bacterial genera isolated here, such as those of the CESP group (*Citrobacter*, *Enterobacter*, *Serratia* and *Providencia*), are producers of inducible AmpC-type β-lactamases, which mediate intrinsic resistance to most third-generation cephalosporins [71]. Intrinsic resistance is increasingly considered relevant mainly when it is detected in opportunistic pathogens. *Stenotrophomonas* sp. has been described as a reservoir and as a vector of acquired ARGs that encode carbapenemases, such as the *bla*_KPC_ gene, especially in aquatic environments [72].

Indeed, ESBL- and carbapenemase-producing Gram-negative bacteria of the Enterobacteriaceae family represent a global threat to human and animal health. These bacteria are the main propagators of the AMR pandemic and are on the World Health Organization priority pathogens list to guide research, discovery, and development of new antimicrobials [73,74].

According to Lapidot and Yaron (2009) [75], contaminated water can increase the ability of pathogens to colonize agricultural crops, favoring resistant bacteria dissemination in the environment and resistant pathogen transmission to humans and animals [76]. Water used in irrigation has already been identified as the likely source of several outbreaks of diseases transmitted by fresh and raw foods, such as fruits and vegetables, which are the most common agricultural crops associated with outbreaks [77].

## 5. Conclusions

This study demonstrates that surface water sources can be considered reservoirs of resistant bacteria and resistance genes in agricultural areas with extensive use of poultry litter as an organic fertilizer. The presence of *sul1*, *sul2*, *intI1* and *intI2* genes and in strains isolated from the studied ponds may indicate the accumulation of resistance to sulfonamides in the environment. The detection of genes encoding ESBLs, mainly *bla*_GES_, which are found less frequently in the environment, also suggests the spread of resistance to β-lactams in the environment. In addition, seasonal influences on the evaluated bacterial communities (mainly periods of high rainfall) are an additional aggravating factor for antimicrobial-resistant bacteria mobilization (soil–water). Considering the extensive use of surface water sources, this study suggests the potential risk of dissemination and transmission of AMR, through different forms of water use, mainly in the irrigation of agricultural crops, which favors the entry of pathogens into the food chain. Therefore, monitoring and understanding of aquatic resistomes under anthropic influence is of great importance to control the environmental spread of AMR. It is still necessary to contain the irrational use of antimicrobials in human medicine, veterinary medicine, and especially in livestock and agriculture.

## Figures and Tables

**Table 1:** Presence of antimicrobial resistance genes in the isolated strains.

Ceftriaxone Resistant Strains	Antimicrobial Resistance Genes			
	*bla*_TEM_		*bla*_SHV_	
*Escherichia* sp. (CCA 1.2) *	1		0	
*Escherichia* sp. (CCA 1.3)	1		0	
*Klebsiella* sp. (CCA 2.1)	1		1	
*Klebsiella* sp. (CCA 2.2)	1		1	
*Klebsiella* sp. (CCA 2.3)	1		1	
**Sulfamethoxazole resistant strains**	*sul1*	*sul2*	*intI1*	*intI2*
*Escherichia* sp. (CSA 1.1) *	0	1	1	0
*Proteus* sp. (CSA 2.1A)	0	1	0	0
*Aeromonas* sp. (CSA 2.2)	0	1	0	0
*Enterobacter* sp. (CSA 2.3)	1	0	1	0
*Enterobacter* sp. (CSA 5.1)	0	0	0	1
*Enterobacter* sp. (CSA 5.2)	0	0	0	1
*Pantoea* sp. (CSB 1.1)	0	0	0	1
*Escherichia* sp. (CSD 1.2)	0	1	0	0
*Escherichia* sp. (CSD 1.3)	0	1	1	0

* The name of the strains corresponds to the origin of their isolation: CCA (CHROMagar containing ceftriaxone and LA), CSA, CSB and CSD (CHROMagar containing sulfamethoxazole and LA, LB and LD, respectively).

## Data Availability

The datasets used and/or analyzed under the current study are available from the corresponding author upon reasonable request.

## Supplementary Materials

The following supporting information can be downloaded at: https://www.mdpi.com/article/10.3390/antibiotics11111650/s1.
