# Supplementary material for: Irrigation Ponds as Sources of Antimicrobial-Resistant Bacteria in Agricultural Areas with Intensive Use of Poultry Litter
Source: Antibiotics (Basel). 2022 Nov 18;11(11):1650. doi: 10.3390/antibiotics11111650 (PMC9686582; doi:10.3390/antibiotics11111650)
Supplement: Supplementary file 1 [file antibiotics-11-01650-s001.zip › antibiotics-1992030-supplementary.pdf]

Supplementary material

Irrigation ponds as sources of antimicrobial resistant bacteria in agricultural areas with intensive use of poultry litter

Eliene S. Lopes[1], Cláudio E.T. Parente[2], Renata C. Picão[3], Lucy Seldin[1*]

[1] Laboratório de Genética Microbiana, Instituto de Microbiologia Paulo de Góes, Universidade Federal do Rio de Janeiro (UFRJ), Rio de Janeiro (RJ), Brazil; [2] Laboratório de Radioisótopos Eduardo Penna Franca, Instituto de Biofísica Carlos Chagas Filho, UFRJ, RJ, Brazil; [3] Laboratório de Investigação em Microbiologia Médica, Instituto de Microbiologia Paulo de Góes, UFRJ, RJ, Brazil.

*Corresponding author: Laboratório de Genética Microbiana, Departamento de Microbiologia Geral, Instituto de Microbiologia Paulo de Góes, Universidade Federal do Rio de Janeiro, Centro de Ciências da Saúde, Bloco I, Ilha do Fundão, CEP 21941-590, Rio de Janeiro, Brazil.

Phone: 55-21-3938.6741

Fax: 55-21-2560.8344

E-mail: lseldin@micro.ufrj.br

Content: Supplementary data include 9 figures and 5 tables.

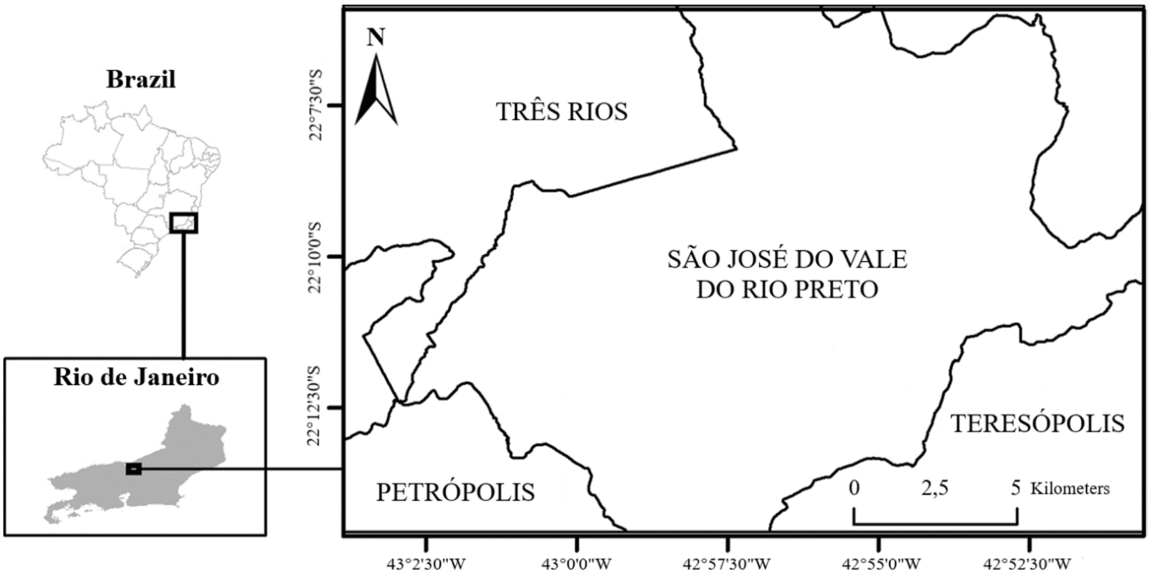

Fig. S1. Study area: municipality of São José do Vale do Rio Preto, located in the upland region of Rio de Janeiro state, in southeastern Brazil.

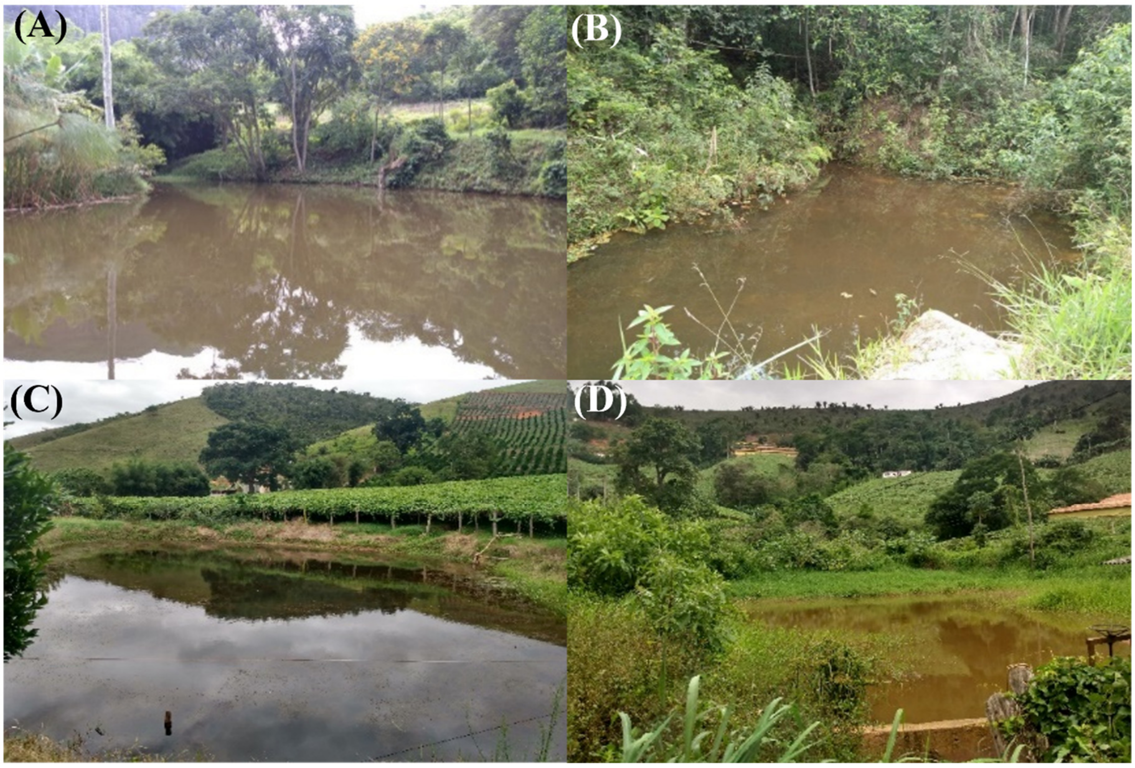

Fig. S2. Ponds around agricultural areas used for crop irrigation: (A) LA (22°19'31.70"W, 42°91'97.11"S) with 245 m^2; (B) LB (22°19'21.71"W, 42°91'84.11"S) with 147 m^2; (C) LC (22°11'46.41"W, 42°95'11.76"S) with 665 m^2 and (D) LD (22°14'92.83"W, 42°.88'17.84"S) with 294 m^2.

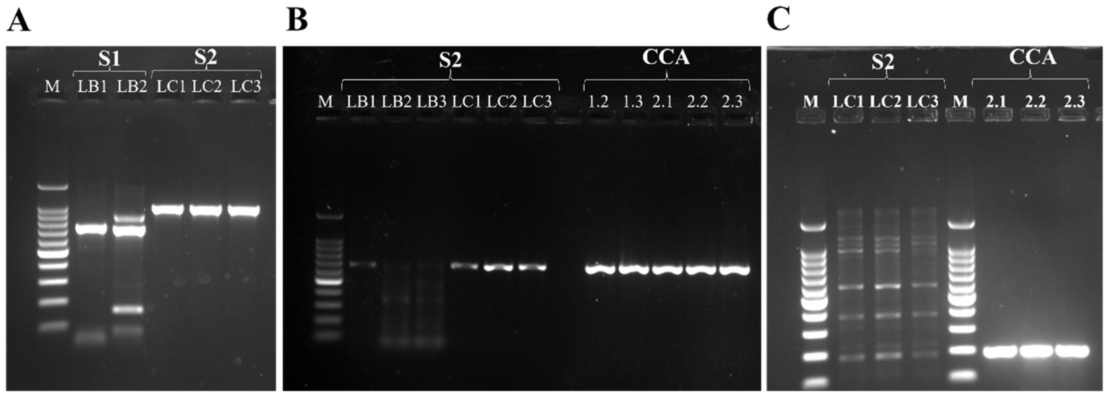

Fig. S3. Agarose gel electrophoresis (1.4%) of the PCR products using the primers for *bla*GES (**A**), *bla*TEM (**B**) and *bla*SHV (**C**) genes. S1 and S2 correspond to the sampling period: high (S1; on March 14, 2019) and low monthly rainfall (S2; on November 25, 2019). Water samples are represented by capital letters followed by the number of replicates: LA (LA1, LA2 and LA3), LB (LB1, LB2 and LB3), LC (LC1, LC2 and LC3) and LD (LD1, LD2 and LD3). CCA corresponds to the ceftriaxone resistant bacterial strains isolated from LA: *Escherichia* sp. (CCA 1.2 and 1.3); *Klebsiella* sp. (CCA 2.1, 2.2 and 2.3). (M) Molecular size marker - 100bp Plus DNA ladder, Promega.

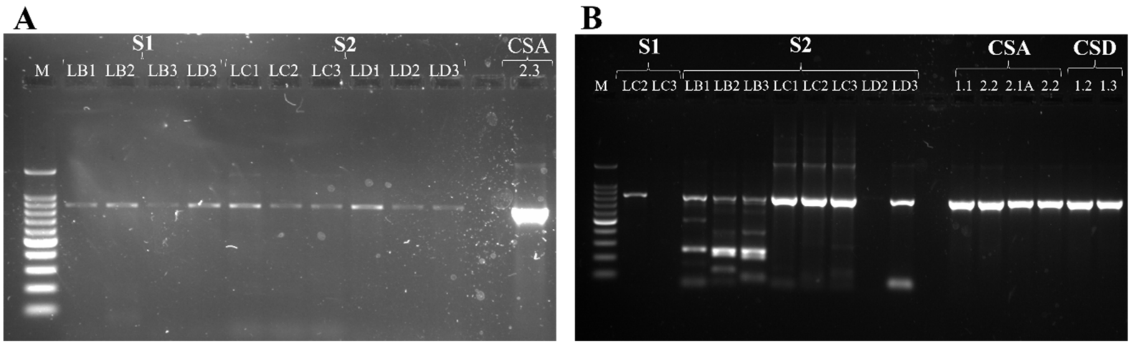

Fig. S4. Agarose gel electrophoresis (1.4%) of the PCR products using the primers for the *sul1* (**A**) and *sul2* (**B**) genes. S1 and S2 correspond to the sampling period: high (S1; on March 14, 2019) and low monthly rainfall (S2; on November 25, 2019). Water samples are represented by capital letters followed by the number of replicates: LA (LA1, LA2 and LA3), LB (LB1, LB2 and LB3), LC (LC1, LC2 and LC3) and LD (LD1, LD2 and LD3). CSA and CSD correspond to the sulfamethoxazole resistant bacterial strains isolated from LA and LD, respectively: *Enterobacter* sp. (CSA 2.3); *Escherichia* sp. (CSA 1.1; CSD 1.2 and 1.3); *Aeromonas* sp. (CSA 2.2); *Proteus* sp. (CSA 2.1A). (M) Molecular size marker - 100bp Plus DNA ladder, Promega.

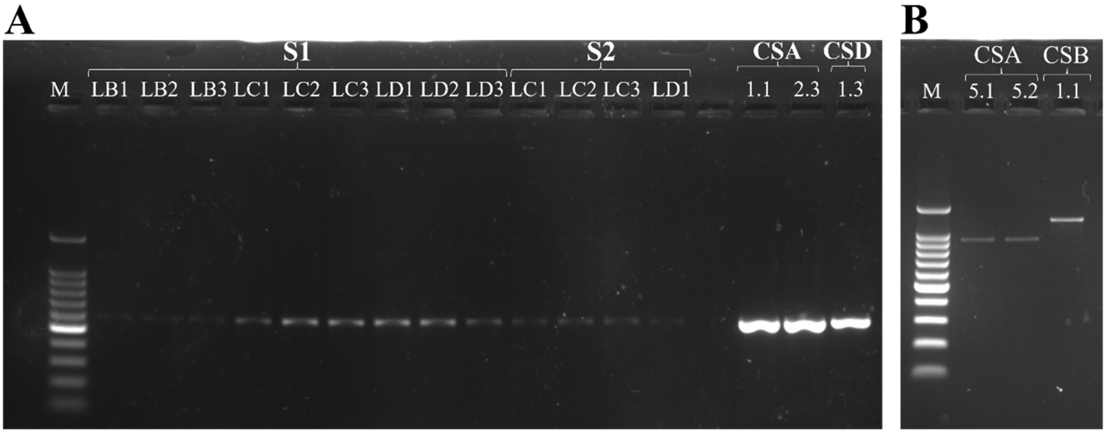

Fig. S5. Agarose gel electrophoresis (1.4%) of the PCR products using the primers for the *intI1* (**A**) and *intI2* (**B**) genes. S1 and S2 correspond to the sampling period: high (S1; on March 14, 2019) and low monthly rainfall (S2; on November 25, 2019). Water samples are represented by capital letters followed by the number of replicates: LA (LA1, LA2 and LA3), LB (LB1, LB2 and LB3), LC (LC1, LC2 and LC3) and LD (LD1, LD2 and LD3). CSA, CSB and CSD correspond to sulfamethoxazole resistant bacterial strains isolated from LA, LB and LD, respectively: *Escherichia* sp. (CSA 1.1; CSD 1.3); *Enterobacter* sp. (CSA 2.3, 5.1 and 5.2); *Pantoea* sp. (CSB 1.1). (M) Molecular size marker - 100bp Plus DNA ladder, Promega.

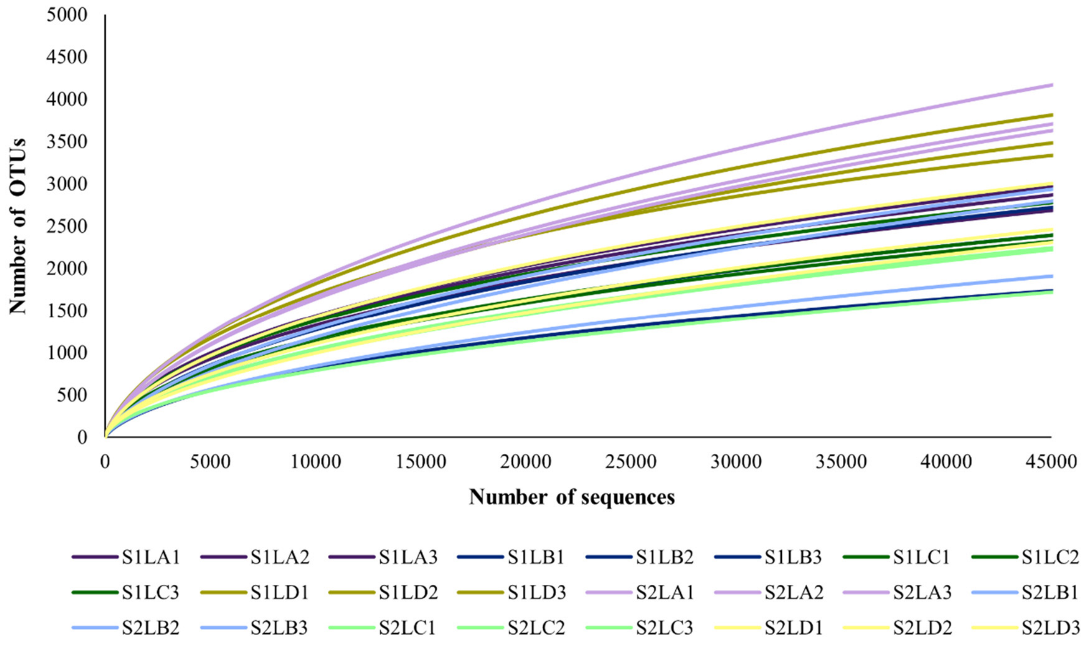

Fig. S6. Individual rarefaction curves of the water samples collected from ponds LA (purple), LB (blue), LC (green) and LD (yellow) in S1 (dark tones) and in S2 (light tones). Replicates are represented by capital letters followed by the number of replicates: LA (LA1, LA2 and LA3), LB (LB1, LB2 and LB3), LC (LC1, LC2 and LC3) and LD (LD1, LD2 and LD3).

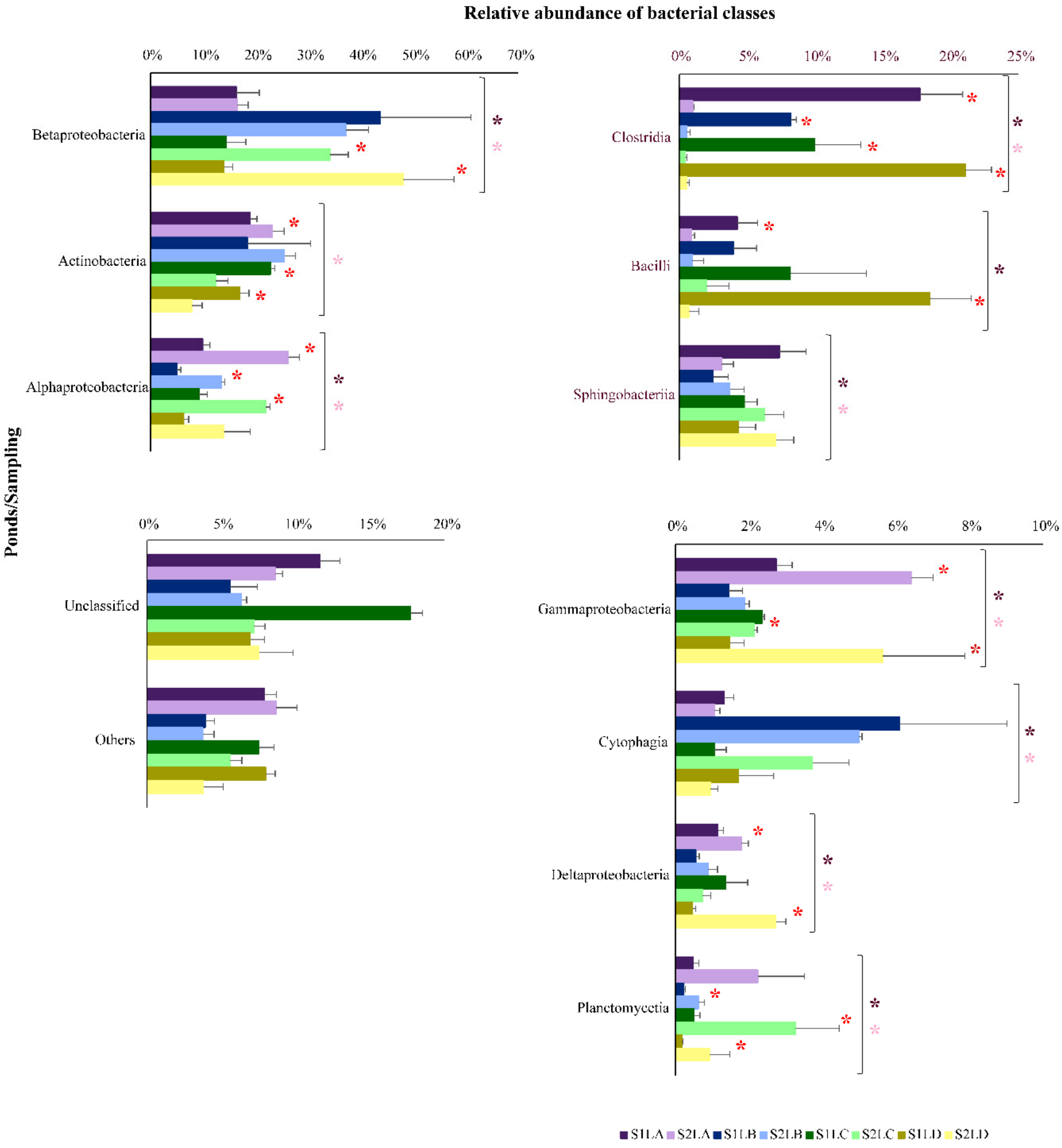

Fig. S7. Relative abundance of bacteria classes in water samples collected in LA (purple), LB (blue), LC (green) and LD (yellow) in S1 (dark tones) and in S2 (light tones). The bars represent the standard deviation. Asterisks (dark pink - S1 and light pink - S2) represent the statistical difference among the ponds in the two samplings (parametric data submitted to a one-way ANOVA and nonparametric data submitted to Kruskal-Wallis). A red asterisk represents the statistical difference between the samplings of each pond (parametric data

submitted to the t test and nonparametric data submitted to Mann-Whitney). The data represented by "others" are formed by more than one taxon and they were not statistically analyzed.

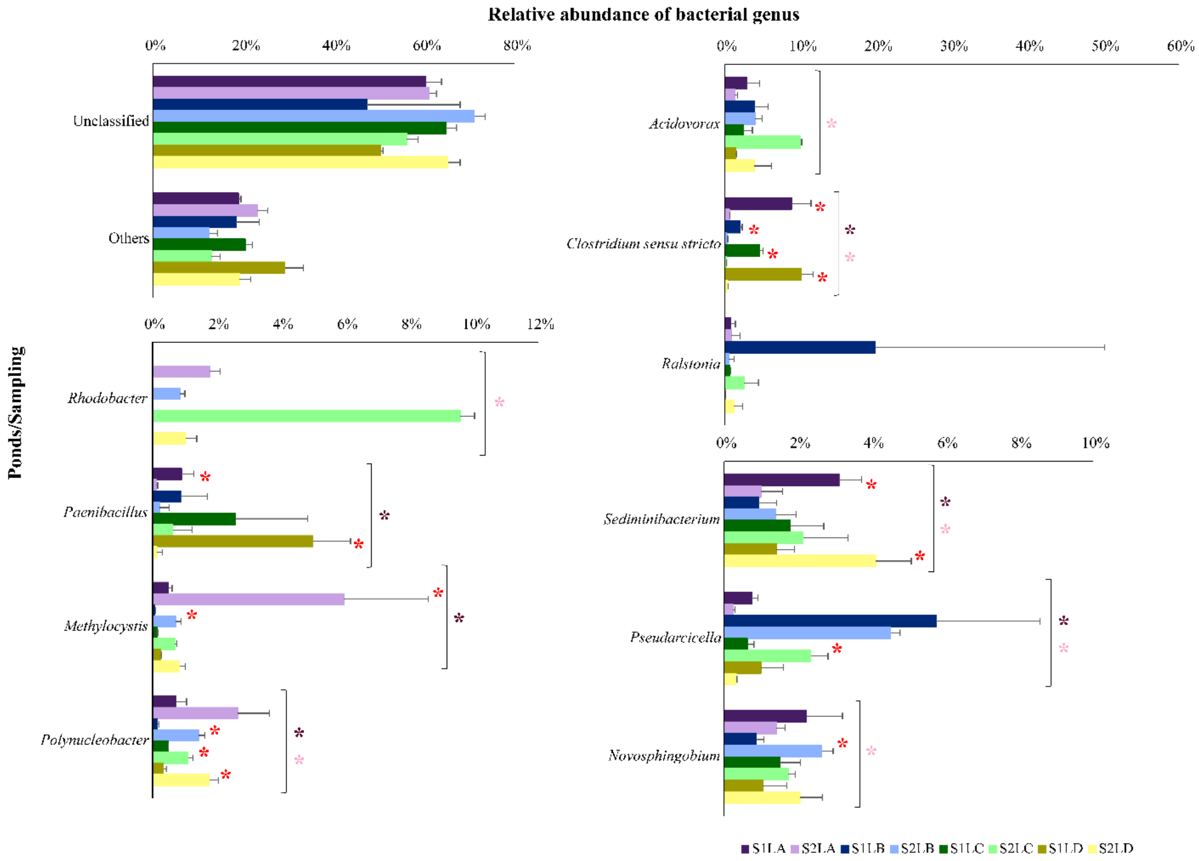

Fig. S8. Relative abundance of bacteria genera in water samples collected in LA (purple), LB (blue), LC (green) and LD (yellow) in S1 (dark tones) and in S2 (light tones). The bars represent the standard deviation. Asterisks (dark pink - S1 and light pink - S2) represent the statistical difference among the ponds in the two samplings (parametric data submitted to a one-way ANOVA and nonparametric data submitted to Kruskal-Wallis). A red asterisk represents the statistical difference between the samplings of each pond (parametric data submitted to the t test and nonparametric data submitted to Mann-Whitney). The data represented by "others" are formed by more than one taxon and they were not statistically analyzed.

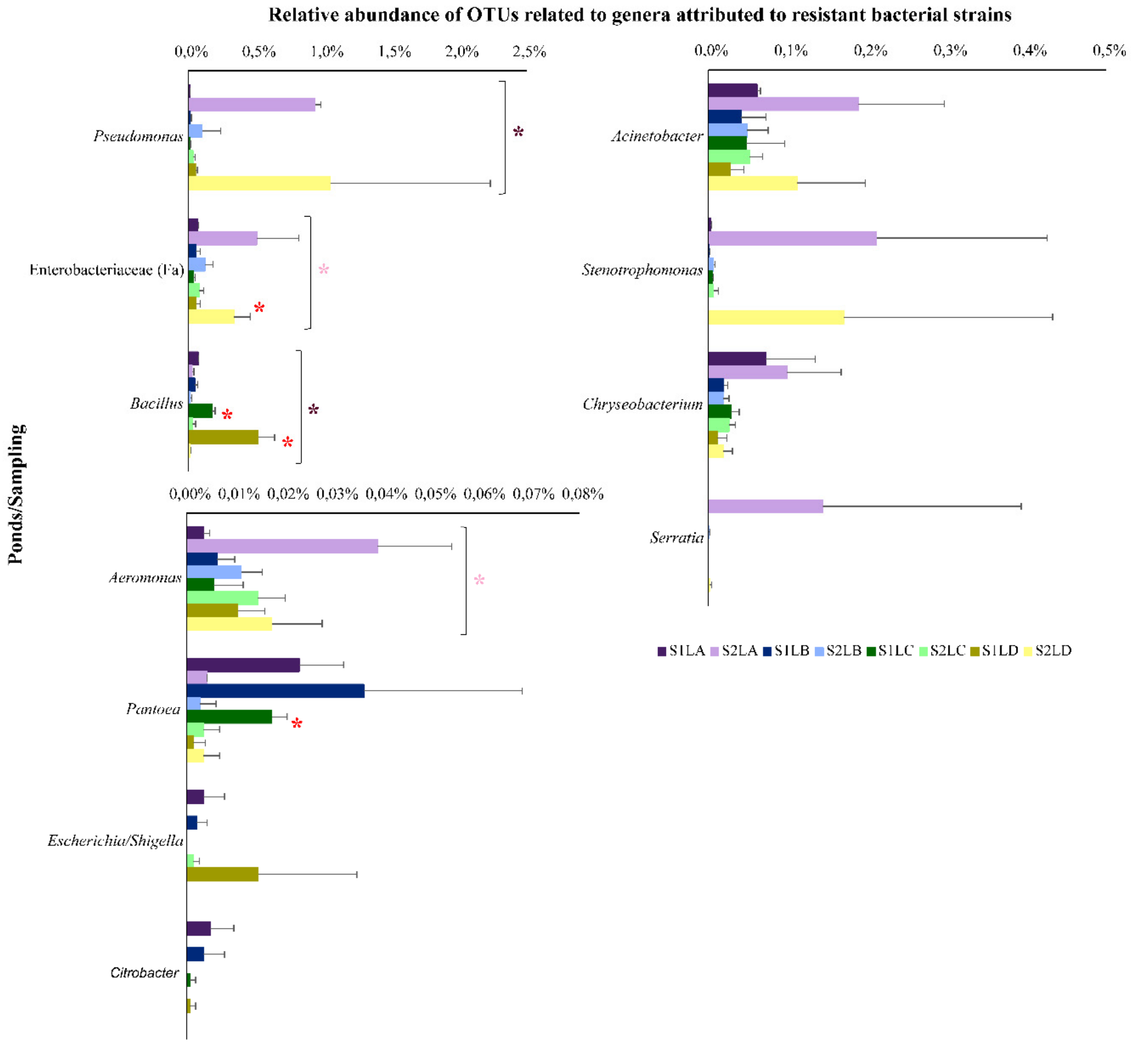

Fig. S9. Relative abundance of OTUs associated with isolated antimicrobial resistant bacterial genera (identified using MALDI-TOF) in water samples collected in LA (purple), LB (blue), LC (green) and LD (yellow) in S1 (dark tones) and in S2 (light tones). The bars represent the standard deviation. Asterisks (dark pink - S1 and light pink - S2) represent the statistical difference among the ponds in the two samplings (parametric data submitted to a one-way ANOVA and nonparametric data submitted to Kruskal-Wallis). A red asterisk

represents the statistical difference between the samplings of each pond (parametric data submitted to the t test and nonparametric data submitted to Mann-Whitney).

Table S1. Physical-chemical characteristics of the water collected from the four ponds. Samplings were in two periods of the year: at the beginning (low volume of rainfall) and at the end of the rainy season (high volume of rainfall).

**Parameters**	**LA**	**LB**	**LC**	**LD**
**First sampling (S1)**				
Salinity (ppt)	0.01	0.01	0.01	0.01
pH	6.5	7.1	7.3	6.9
Temperature (°C)	24	23.7	32.8	27.3
**Second sampling (S2)**				
Salinity (ppt)	0.01	0.01	0.02	0.01
pH	7.2	7.2	10.1	7.4
Temperature (°C)	21.2	20.4	27.5	24.3

Table S2: Sequence of primers used for the amplification of antimicrobial resistance genes and genes encoding integrases

**Gene**	**Primers**	**Sequence (5'-3')**	**Reference**	**Amplification conditions**
intI1	intM1-UF<br>intM1-DR	ACGAGCGCAAGGTTTCGGT<br>GAAAGGTCTGGTCATACATG	[27]	94ºC - 10 min; 30 X (94º - 30 sec; 53º - 30 sec; 72º - 2 min); 72º - 7min
intI2	intM2-UF<br>intM2-DR	GTGCAACGCATTTTGCAGG<br>CAACGGAGTCATGCAGATG		
bla$_{\text{CTX-M-½}}$	mCTX-1/2-F<br>mCTX-1/2-R	ATGTGCAGYACCAGTAA<br>CGCTGCCGGTTTTATCSCCC	[28,29]	95ºC - 10 min; 30 X (95º - 30 sec; 55º - 30 sec; 72º - 45 sec); 72º - 10 min
bla$_{\text{CTX-M-8}}$	mCTX-8-F<br>mCTX-8-R	AACRCRCAGACGCTCTAC<br>TCGAGCCGGAASGTGTYAT		
bla$_{\text{CTX-M-14}}$	mCTX-14-F<br>mCTX-14-R	GGTGACAAAGAGARTGCAACGGAT<br>TTACAGCCCTTCGGCGATGA		
bla$_{\text{SHV}}$	mSHV-F<br>mSHV-R	CTTGACCGCTGGGAAACGG<br>AGCACGGAGCGGATCAACGG		
bla$_{\text{TEM}}$	mTEM-F<br>mTEM-R	CCCTTATTCCCTTTYTTGCGG<br>AACCAGCCAGCCWGAAGG		
bla$_{\text{GES}}$	mGES-F<br>mGES-R	AGCAGCTCAGATCGGTGTTG<br>CCGTGCTCAGGATGAGTTG		
bla$_{\text{MOX-1}}$, *bla*$_{\text{MOX-2}}$, *bla*$_{\text{CMY-1}}$, *bla*$_{\text{CMY-8 a CMY-11}}$	MOXM-F<br>MOXM-R	GCTGCTCAAGGAGCACAGGAT<br>CACATTGACATAGGTGTGGTGC	[30]	94ºC - 3 min; 25 X (94º - 30 sec; 64º - 30 sec; 72º - 1 min); 72º - 7 min
bla$_{\text{CMY-2 a CMY-7}}$, *bla*$_{\text{CMY-31}}$	CITM-F<br>CITM-R	TGGCCAGAACTGACAGGCAAA<br>TTTCTCCTGAACGTGGCTGGC		
bla$_{\text{DHA-1}}$, *bla*$_{\text{DHA-2}}$	DHAM-F<br>DHAM-R	AACTTTCACAGGTGTGCTGGGT<br>CCGTACGCATACTGGCTTTGC		
bla$_{\text{ACC}}$	ACCM-F<br>ACCM-R	AACAGCCTCAGCAGCCGGTTA<br>TTCGCCGCAATCATCCCTAGC		
bla$_{\text{MIR}}$, *bla*$_{\text{ACT}}$	EBCM-F<br>EBCM-R	TCGGTAAAGCCGATGTTGCGG<br>CTTCCACTGCGGCTGCCAGTT		
bla$_{\text{FOX-1 a FOX-5b}}$	FOXM-F<br>FOXM-R	AACATGGGGTATCAGGGAGATG<br>CAAAGCGCGTAACCGGATTGG		

sul1	Sul1-F<br>Sul1-R	GAATAAATCGCTCATCATTTTCGG<br>CGAATTCTTGCGGTTTCTTTCAGC	[31]	95ºC - 10 min; 30 X (95º - 1 min; 52º - 45 sec; 72º - 1 min); 72º - 1min
sul2	Sul2-F<br>Sul2-R	ATGGTGACGGTGTTCGGCATTCTGA<br>CTAGGCATGATCTAACCCTCGGTCT	[31]	95ºC - 10 min; 30 X (95º - 1 min; 55º - 45 sec; 72º - 1 min); 72º - 1min
qnrA	QnrAm-F<br>QnrAm-R	AGAGGATTTCTCACGCCAGG<br>TGCCAGGCACAGATCTTGAC	[32]	95ºC - 10 min; 25 X (95º - 45 sec; 58º - 45 sec; 72º - 15 sec); 72º - 3 min
qnrS	QnrSm-F<br>QnrSm-R	GCAAGTTCATTGAACAGGGT<br>TCTAAACCGTCGAGTTCGGCG		
qnrB	QnrBm-F<br>QnrBm-R	GGMATHGAAATTCGCCACTG*<br>TTTGCYGYYCGCCAGTCGAA*		
qnrC	QnrCm-F<br>QnrCm-R	GCGAATTTCCAAGGGGCAAA<br>ACCCGTAATGTAAGCAGAGCAA	[33]	95ºC - 10 min; 25 X (95º - 45 sec; 58º - 45 sec; 72º - 15 sec); 72º - 3 min
qnrD	QnrDm-F<br>QnrDm-R	AGGTGTAGCATGTATGGAAAAGC<br>ACATTGGGGCATTAGGCGTT		
qnrVC	QnrVCm-F<br>QnrVCm-R	GAGYTKTATGGTTTAGAYCCTCG*<br>TGTTCYTGYTGCCACGARCA*		
qepA	QepA-F<br>QepA-R	GCAGGTCCAGCAGCGGGTAG<br>CTTCCTGCCCGAGTATCGTG	[34]	95ºC - 10 min; 25X (95 ºC - 45 sec; 58 ºC por 45 sec; 72 ºC - 15 sec); 72º - 15 min.

*M = A or C; H = A or C or T; Y = C or T; K = T or G; R = A or G

Table S3: Identification of strains grown on CHROMagar supplemented with 50 µg/ml ciprofloxacin

Genera	LA	LB	LC	LD
Escherichia sp.	0*	0	0	3

* Number of strains identified using MALDI-TOF

Table S4: Identification of strains grown on CHROMagar supplemented with 8 µg/ml ceftriaxone

Genera	LA	LB	LC	LD
Acinetobacter sp.	0*	0	5	0
Bacillus sp.	0	2	4	4
Chryseobacterium sp.	2	6	6	1
Elizabethkingia sp.	0	3	0	0
Enterobacter sp.	0	0	0	1
Escherichia sp.	3	0	0	3
Klebsiella sp.	3	0	1	0
Proteus sp.	0	0	0	1
Pseudomonas sp.	2	0	3	0
Stenotrophomonas sp.	0	0	4	0

* Number of strains identified using MALDI-TOF

Table S5: Identification of strains grown on CHROMagar supplemented with 60 µg/ml sulfamethoxazole

**Genera**	**LA**	**LB**	**LC**	**LD**
Aeromonas sp.	2*	0	0	0
Bacillus sp.	0	1	0	2
Citrobacter sp.	0	0	2	1
Cronobacter sp.	1	0	0	0
Enterobacter sp.	4	0	1	1
Escherichia sp.	1	1	2	2
Klebsiella sp.	1	1	1	2
Pantoea sp.	0	1	1	0
Proteus sp.	2	0	1	1
Pseudomonas sp.	3	0	6	0
Serratia sp.	0	4	0	0

* Number of strains identified using MALDI-TOF

References

27. Xu, X.; Kong, F.; Cheng, X.; Yan, B.; Du, X.; Gai, J.; Ai, H.; Shi, L.; Iredell, J. Integron Gene Cassettes in *Acinetobacter* spp. Strains from South China. *Int. J. Antimicrob. Agents* **2008**, 32, 441–445, doi:10.1016/j.ijantimicag.2008.05.014.
28. Campana, E.H.; Xavier, D.E.; Petrolini, F.V.B.; Cordeiro-Moura, J.R.; Araujo, M.R.E. de; Gales, A.C. Carbapenem-Resistant and Cephalosporin-Susceptible: A Worrisome Phenotype among *Pseudomonas aeruginosa* Clinical Isolates in Brazil. *Braz. J. Infect. Dis.* **2017**, 21, 57–62, doi:10.1016/j.bjid.2016.10.008.
29. Picão, R.C.; Poirel, L.; Gales, A.C.; Nordmann, P. Diversity of β-Lactamases Produced by Ceftazidime-Resistant *Pseudomonas aeruginosa* Isolates Causing Bloodstream Infections in Brazil. *Antimicrob. Agents Chemother*. **2009**, 53, 3908–3913, doi:10.1128/AAC.00453-09.
30. Pérez-Pérez, F.J.; Hanson, N.D. Detection of Plasmid-Mediated AmpC β-Lactamase Genes in Clinical Isolates by Using Multiplex PCR. *J. Clin. Microbiol.* **2002**, 40, 2153–2162, doi:10.1128/JCM.40.6.2153-2162.2002.
31. Toleman, M.A.; Bennett, P.M.; Bennett, D.M.C.; Jones, R.N.; Walsh, T.R. Global Emergence of Trimethoprim/Sulfamethoxazole Resistance in *Stenotrophomonas maltophilia* Mediated by Acquisition of *sul* Genes. *Emerg. Infect. Dis.* **2007**, 13, 559–565, doi:10.3201/eid1304.061378.
32. Cattoir, V.; Poirel, L.; Rotimi, V.; Soussy, C.J.; Nordmann, P. Multiplex PCR for Detection of Plasmid-Mediated Quinolone Resistance Qnr Genes in ESBL-Producing Enterobacterial Isolates. *J. Antimicrob. Chemother.* **2007**, 60, 394–397, doi:10.1093/jac/dkm204.
33. Kraychete, G.B.; Botelho, L.A.B.; Campana, E.H.; Picão, R.C.; Bonelli, R.R. Updated Multiplex PCR for Detection of All Six Plasmid-Mediated Qnr Gene Families. *Antimicrob. Agents Chemother.* **2016**, 60, 7524–7526, doi:10.1128/AAC.01447-16.
34. Yamane, K.; Wachino, J.I.; Suzuki, S.; Kimura, K.; Shibata, N.; Kato, H.; Shibayama, K.; Konda, T.; Arakawa, Y. New Plasmid-Mediated Fluoroquinolone Efflux Pump, QepA, Found in an *Escherichia coli* Clinical Isolate. *Antimicrob. Agents. Chemother.* **2007**, 51, 3354–3360, doi:10.1128/AAC.00339-07.
